# Mid-term Clinical Outcome of Microvascular Gracilis Muscle Flaps for Defects of the Hand

**DOI:** 10.1007/s00402-024-05207-7

**Published:** 2024-01-24

**Authors:** Tatjana Pastor, Rahel Meier, Dominique Merky, Luzian Haug, Torsten Pastor, Cédric Zubler, Esther Vögelin

**Affiliations:** 1 Department for Plastic and Hand Surgery, Inselspital University Hospital Bern, University of Bern, Bern, Switzerland; 2 AO Research Institute Davos, Davos, Switzerland; 3 Department of Orthopedic and Trauma Surgery, Lucerne Cantonal Hospital, Lucerne, Switzerland

**Keywords:** Gracilis muscle flap, Covering hand defects, Soft tissue defects hand, Hand surgery, Microsurgery

## Abstract

Purpose: Gracilis muscle flaps are useful to cover defects of the hand. However, there are currently no studies describing outcome measurements after covering soft tissue defects using free flaps in the hand. Aim: To analyze mid-term results of gracilis muscle flap coverage for defects on the hand, with regard to functional and esthetic integrity. Methods: 16 patients aged 44.3 (range 20–70) years were re-examined after a mean follow-up of 23.6 (range 2–77) months. Mean defect size was 124 (range 52–300) cm^2^ located palmar (*n* = 9), dorsal (*n* = 6), or radial (*n* = 1). All flaps were performed as microvascular muscle flaps, covered by split thickness skin graft. Results: Flaps survived in 15 patients. 6 patients required reoperations. Reasons for revisions were venous anastomosis failure with total flap loss (*n* = 1) requiring a second gracilis muscle flap; necrosis at the tip of the flap (*n* = 1) with renewed split thickness skin cover. A surplus of the flap (*n* = 2) required flap thinning and scar corrections were performed in 2 patients. Mean grip strength was 25% (range 33.3–96.4%) compared to the contralateral side and mean patient-reported satisfaction 1.4 (range 1–3) (1 = excellent; 4 = poor). Conclusions: Gracilis muscle flaps showed a survival rate of 94%. Patients showed good clinical outcomes with acceptable wrist movements and grip strength as well as high reported satisfaction rates. Compared to fasciocutaneous free flaps, pliability and thinness especially on the palmar aspect of the hand are advantageous. Hence, covering large defects of the hand with a gracilis muscle flap can be a very satisfactory procedure.

Level of evidence: IV observational.

## Introduction

Treatment of patients with soft tissue defects of the hands is surgically challenging and commonly encountered in the setting of trauma or infections. In patients with moderate or large size defects, which are not coverable with local flaps, free tissue transfer is considered. Primary treatment goal is the restoration of the functionality of the hand. This requires thin, pliable skin, which permits mobility of joints, allows gliding of the underlying tendons, and offers a vascular bed for healing of fractured bones. However, nowadays, also the esthetic outcome shifts more into the focus of the literature [1]. The gracilis muscle flap was first described by Harii, Ohmori and Torii in 1976 [2] and is a very versatile flap in reconstructive microsurgery. Based on the indication, it may be used as a free flap for distant reconstructions and the advantages have already been described in a variety of clinical reports [3–6]. Moreover, it is already successfully used as a free functional flap in upper extremity reconstruction [7–10]. Nevertheless, there are concerns on the bulky appearance of the flap when it is harvested as a myocutaneous flap. Using a free gracilis muscle flap covered with split thickness skin graft, the alignment and the appearance of the hand are more appealing. However, detailed outcome reports of gracilis muscle flaps used for covering large soft tissue defects of the hand are missing in the current literature. It was therefore the purpose of this study to (1) analyze clinical mid-term results of free gracilis muscle flaps with skin grafts for extensive soft tissue defects of the hand considering the function of the hand and patient satisfaction and (2) to assess reasons for postoperative complications, such as flap necrosis, tendon adhesions, flap bulkiness, and scar contraction.

## Materials and methods

### Ethical approval

Ethical approval for this retrospective study was granted by the responsible institutional review board BASEC Nr. 2021–01346 and all included patients provided written consent for the study.

### Patients

Between February 2006 and November 2020, 16 consecutive patients (10 men, 6 women) underwent microvascular gracilis muscle free flap coverage for large soft tissue defects on their hands. The mean age was 44 years (range 20–70 years) at the time of the flap harvest. The dominant hand was affected in 7 cases (44%). All but one patient (94%) underwent surgical emergency intervention prior to gracilis muscle flap coverage. In this single patient, who refused medical treatment after the initial trauma, wound healing was achieved by secondary intention including an inferior functional and esthetic result. 11 of 16 patients (69%) received initially a vacuum dressing therapy. Surgical intervention other than debridement included fasciotomy due to an impending compartment syndrome in 5 patients (31%); release of carpal tunnel in 4 patients (25%); release of Guyon’s canal as well as an amputation of the 3rd digit in 1 patient (6%); tangential resection of the proximal carpal row and 8 cm of the distal radius due to osteomyelitis in 1 patient; resection of superficial flexor tendon (FDS), metacarpophalangeal joint (MCPJ) resection of the 5th digit in 1 patient; resection of the 2nd digit at MCPJ level in 1 patient; resection of 2nd and 5th digit at level of the metacarpal bone (MC) as well as shortening of the deep flexor digitorum (FDP) and FDS tendon in 1 patient; carpal stabilization with k-wires, refixation of the scapho-lunate (SL) ligament after a perilunate luxation as well as resection of the extensor indicis (EIP) tendon in 1 patient. Another patient received an osteosynthesis at the distal radius (3.5 LCP 6-hole) and the distal ulna (3.5 LCP 10-hole), flexor pollicis longus (FPL) tendon reconstruction with FDP, as well as an opponens plasty with FDS IV. Furthermore, a reconstruction of the palmar carpometacarpal (CMC) ligaments with 2 mini anchors as well as an amputation of 2nd digit. Another patient amputated all his digits including the thumb in a mechanic press. His thumb as well as digits 2, 3, and 5 could be replanted by means of vascular and interpositional nerve grafts. The last patient lost his thumb during an electric burn accident and refused medical treatment for several years after the initial trauma due to an underlying psychological disorder.

Mean defect size in all patients was 124 cm^2^ (range 52–300 cm^2^) and was located at the palmar aspect of the hand (*n* = 9), at the dorsal aspect of the hand (n = 6) or at the radial aspect of the hand (*n*=1). See Table 1 for a detailed description of all enrolled patients, injury patterns, and treatments.

**Table 1:** Detailed description of all enrolled patients, injury patterns, and treatments

No	Type of lesion	Mean (range) defect size (cm^2^)	Primary treatment	Secondary reconstruction [Mean (range) days after injury]
1	Infection after i.v. drug abuse	180	Wound healing by secondary intention	28
11	Agriculturally contaminated wounds or infection	128 (72–300)	Vacuum treatment	12 (5—19)
5	Severe crush injuries or infection	144 (56–300)	Fasciotomy due to impeding compartment syndrome	8 (4—16)
4	Severe crush injuries	139 (52–300)	Release of carpal tunnel	16 (12–19)
1	Crush injury	300	Release of loge de Guyon	7
1 1 2 2	Severe crush injuries or open fractures	108 (52–300)	Amputation of 3rd digit Amputation of 5th digit Amputation of 2nd digit Amputation of 2nd and 5th digit	13 (4–19)
1	Osteomyelitis	72	Tangential resection of proximal row and distal radius of 8 cm	3
2	Severe crush injuries	74 (52–96)	Resection of FDS Shortening of FDS and FDP	11 (6–16)
1	Perilunate luxation	110	Carpal stabilization with k-wires, refixation of the scapho-lunate (SL) ligament; resection of EIP	7
1	Milling injury with osteocutaneous defect5	56	2nd ray resection, forearm osteosynthesis; FPL reconstruction with FDP II; Opponensplasty with FDS IV; palmar CMC I ligament reconstruction	4
1	Amputation of Dig 1–5 in a mechanic press	161	Replantation of 2nd, 3rd and 5th digit	5
1	Electric burn and thumb loss	104	N555o initial treatment	535

*i.v.* intravenous, *Dig*. Digit, *FDS* Flexor digitorum superficialis, *FDP* Flexor digitorum profundus, *EIP* Extensor indices proprius

### Reasons for soft tissue defects

10 patients (63%) needed coverage of large soft tissue defects after a major trauma to their hands. The remaining 6 patients were referrals to our institution due to severe infections. 1 (6%) patient injected petroleum into his soft tissues due to an underlying psychologic disorder; 1 patient was abusing intravenous (i.v.) drugs and suffered large infections on both hands; another patient had a wound infection after resection of a leiomyosarcoma; the remaining 3 patients had severe infections after minor lesions to their hands. Microbiology showed group A beta hemolytic streptococci in 5 patients (31%); *Enterococcus cloacae* in 1 patient, *Streptococcus milleri* in 1 patient, and bacillus cereus as well as methicillin-resistant *Staphylococcus aureus* (MRSA) in another patient. Two patients (13%) suffered from diabetes type 2, 10 (62%) were smokers with an average of 14 pack years (range 5–40 pack years) and 2 patients reported continuous i.v. drug abuse. Average body mass index (BMI) was 25 kg/m^2^ (range 21–36 kg/m^2^) with 2 patients over 30 kg/m^2^.

### Surgical technique

Surgery at our institution is performed in two teams as described by Harii et al. [2]; one team harvests the flap while a second team prepares the vessels at the recipient site. The pedicle at its origin is divided when the recipient site is ready to insert the flap. The origin of the pedicle is ligated before performing the division of the gracilis muscle’s origin at the ramus of the pubis for protection purposes. Afterward, one team closes the donor site at the thigh while the other team performs microsurgery on two veins and one artery under a microscope. Reinnervation of the flap is only required in functional flap transfers but not in a soft tissue defect reconstruction at the hand or forearm. However, the obturator nerve can be followed up proximally all the way to its emergence from the main trunk and it might be used as a nerve graft to simultaneously reconstruct a nerve defect at the recipient site, which was not performed in the patients of the current study. A meshed split thickness skin graft is collected and applied to the muscle flap after the blood flow to the muscle flap has been restored.

### Clinical assessment

Clinical outcome parameters included pain, satisfaction, work capacity, sports participation, complications, and reoperations. Clinical examination included assessment of active range of motion measurements of the wrist, all MCP joints as well as the opposition of the thumb according to the Kapandji score [12]. The Jamar® hydraulic hand dynamometer (J. A. Preston Corporation, Clifton, NJ) was used for measuring grip strength in both hands of each participant. Each patient was instructed in the correct handling of the instrument with an upright straight sitting position, a 90° flexion of the elbow, the upper arm in a neutral position, the wrist was held at 0 to 30° extension, and the forearm in neutral position with no resting on a surface of the forearm or the hand. Patients were additionally assessed using the SF-36 [13] as well as the visual analog scale (VAS) [14] and rated their subjective functional outcome (SFO), cosmetic outcome (SCO) and sensibility outcome (SSO) on a scale from 0 to 100. Additionally, patients rated their overall postoperative satisfaction as “excellent”, “good”, “fair”, or “unsatisfactory”.

### Statistical analysis

Descriptive statistical analysis was performed with SPSS software package (IBM SPSS Statistics, V23, IBM, Armonk, NY) with calculation of mean and range for all reported values.

## Results

### Complications and reoperations

Microvascular gracilis muscle flaps survived in 15 patients (94%). In one patient, an early venous anastomosis failure occurred and due to late reporting, flap loss occurred, and the opposite gracilis muscle flap from the contralateral side was harvested. This flap survived. Further complications occurred later on in 5 patients (31%) at a mean of 407 days (range, 367–446 days) after gracilis muscle flap harvesting and applied split thickness skin grafting. Reasons for late revisions were a surplus of the flap (n = 2); scar contraction (n = 2) and necrosis at the tip of the flap (n = 1), see Table 2 for detailed description of complications.

**Table 2:** Individual description of late graft complications (5 of 16 patients (31%))

No	Sex	Age at surgery	Reason for gracilis flap	Days to Complication	Complication	Reoperation	Ultimately flap survival
1	F	42	Infection after milling injury	404	Scar shrinkage	Z-plastic interdigital 1,2	Yes
2	F	34	Infection after minor finger injury	367	Flap surplus	Flap shrinkage	Yes
3	M	46	Infection after minor finger injury	441	Flap surplus and FCR attachments	Flap shrinkage and FCR tenolysis	Yes
4	M	58	Crush injury with imminent compartment syndrome	420	Necrosis at tip of the flap 4 × 5 cm	Debridement and renewed split skin coverage	Yes
5	M	24	Crush injury with amputation of dig 2–4 and thumb	446	Scar shrinkage	Z-plastic palmar wrist	Yes

*FCR* flexor carpi radialis tendon, *F* female, *M* male, Interdigital 1,2: between thumb and index finger

Two patients (13%) needed flap thinning; 2 more patients required corrections of their scars, and in one patient, debridement and a renewed split thickness skin grafting for covering the muscle was required.

### Clinical outcome

At final follow-up, 1 (6%) patient had moved abroad, and 5 (31%) patients were deceased and thus unavailable for final follow-up, leaving 10 out of 16 patients for re-examination after a mean follow-up of 28.3 (range 6–77) months. Patient-reported outcome parameters, such as subjective outcome SFO, SCO, SSO, satisfaction, and SF-36 score, could therefore only be obtained for those patients (*n* = 10). However, chart review revealed no documented postoperative complications and the microvascular gracilis muscle flap survived in the remaining 6 patients until the latest follow-up at our institution (mean 13 (range 2–41) months). The other outcome parameters could be obtained from chart review for these patients. Furthermore, 9 fingers in 7 patients have been amputated either during initial trauma or during the initial extended debridement; thus, function of these digits could not be examined up to final follow-up.

**Fig. 1:**
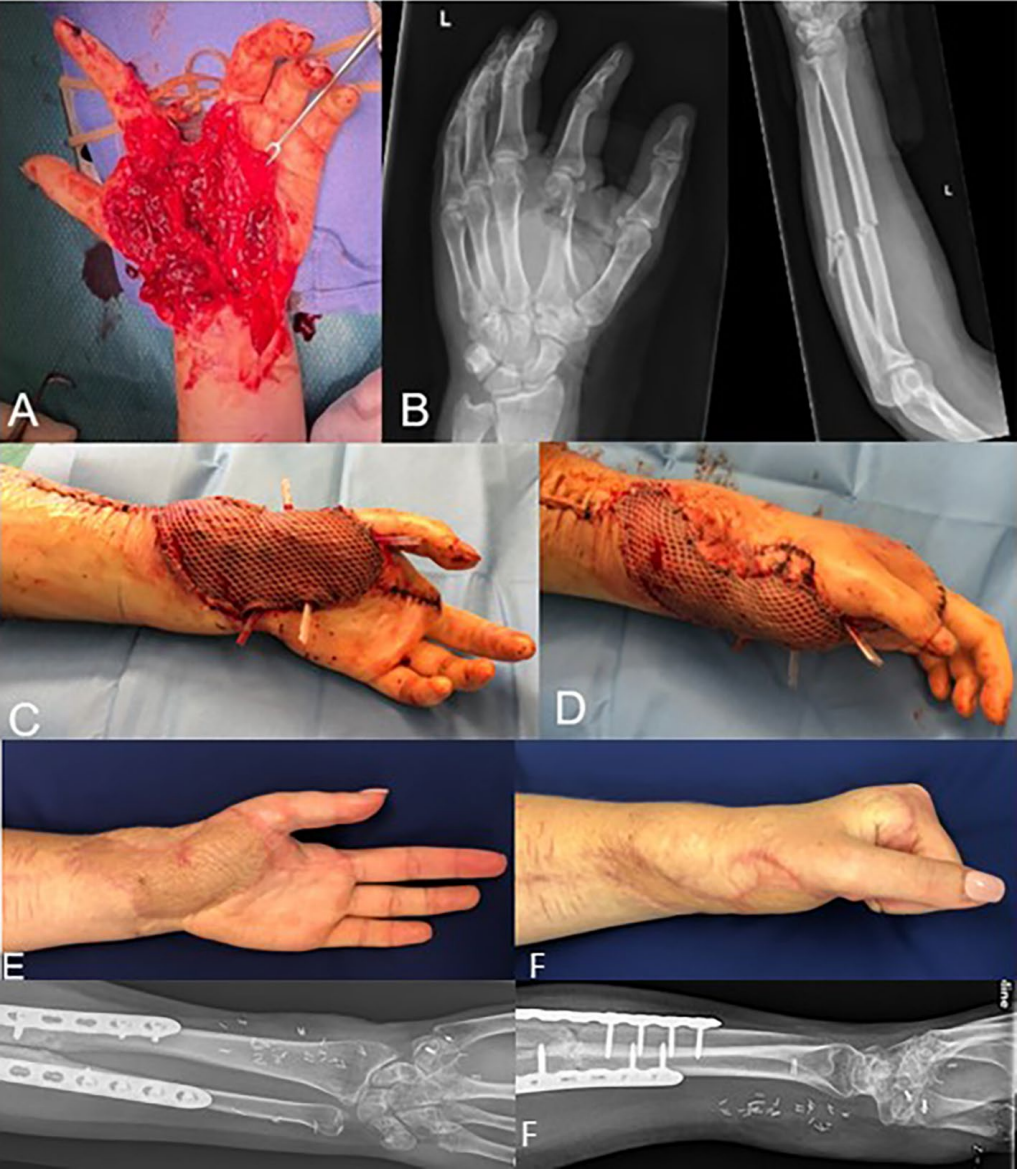
20-year-old female with severe devascularising milling injury with deep osteotendocutaneous defect zone, which had led to an index ray amputation and pronounced soft tissue defect (A–B). The size of the defect was 56 cm^2^, mainly palmar and crossing the wrist joint. Initial treatment included osteosynthesis of the forearm, soft tissue reconstruction of tendons and nerves as well as vessels and ligaments. After 4 days, a microvascular gracilis muscle flap including a meshed split thickness skin graft was performed (C – D). Pictures E – F demonstrate the clinical and radiological follow-up of 9 months after surgery. She rated her subjective cosmetically and functional outcome with 85% and her overall satisfaction rate was 1 (excellent)

**Fig. 2:**
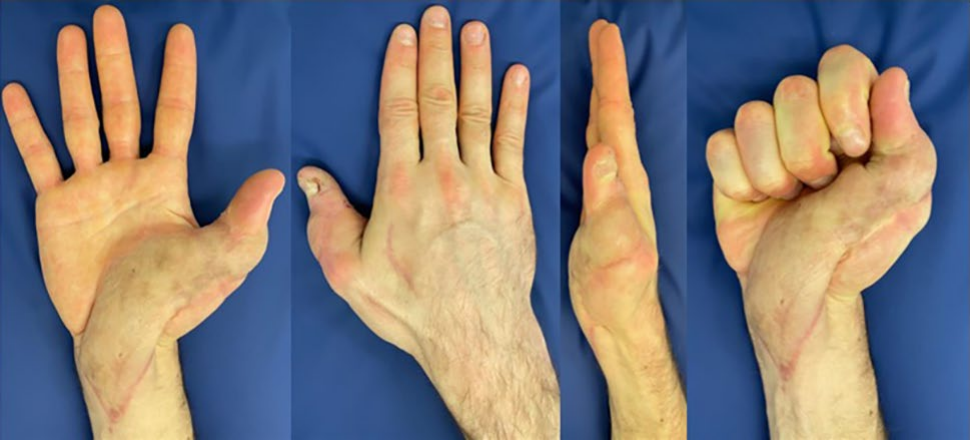
Outcome after a microvascular gracilis muscle flap including a meshed split thickness skin graft in a 34-year-old male after an infection

**Fig. 3:**
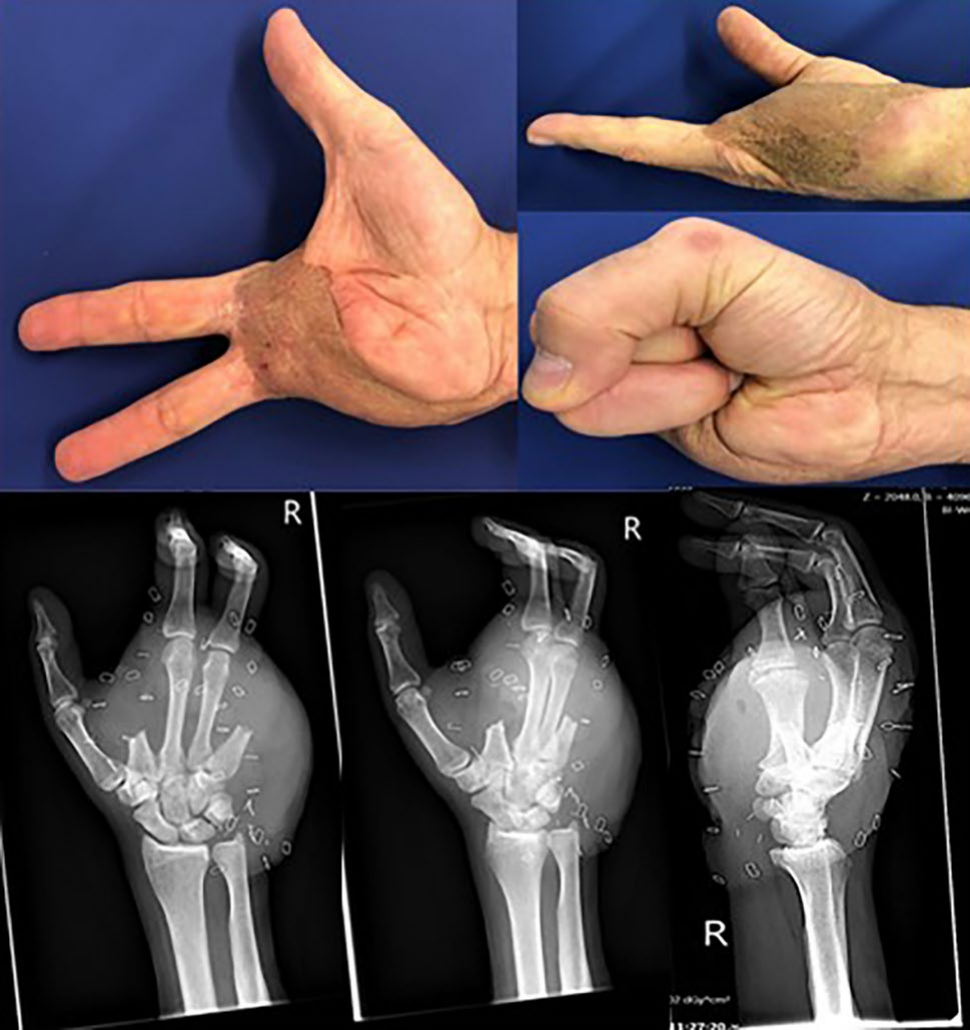
Outcome of a 56-year-old male after a motorcycle accident and ray amputation of 2nd and 5th digit

7 out of 16 patients (44%) were working full-time at the time of final follow-up, but 1 of those patients (6%) had undergone a re-education from a laboring to a non-laboring profession. Of the remaining 9 patients (66%), 7 (44%) had a 100% permanent incapacity for work and 2 (13%) had a partial (50%) incapacity for work. All 9 patients received workers’ compensations payments. Clinical findings and patient-reported outcome parameters at final follow-up are displayed in Table 3. Clinical pictures of 3 patients are demonstrated in Figures 1, 2, and 3.

**Table 3:** Clinical findings and patient-reported outcome parameters at final follow-up

Variable	*n*	Mean / Median	(Range) / SD
VAS, pts	10	0.9 / 0.0	(0.0–4.0) / 1.5
SF-36 score absolute, pts (149)	10	97.6 / 97.5	(91.0–104.0) / 5.9
Physical functioning, %	10	84.0 / 87.5	(70.0–100.0) / 12.9
Limitations (physical health), %	10	50.0 / 50.0	(0.0–100.0) / 47.1
Limitations (emotional health), %	10	93.3 / 100.0	(33.3–100.0) / 21.1
Energy/fatigue, %	10	60.0 / 55.0	(40.0–85.0) / 19.3
Emotional well-being, %	10	80.0 / 84.0	(56.0–96.0) / 12.8
Social functioning, %	10	88.8 / 100.0	(50.0–100.0) / 19.0
Pain, %	10	77.0 / 78.8	(22.5–100.0) / 23.2
General health, %	10	83.5 / 87.5	(50.0–100.0) / 15.6
Health change, %	10	60.0 / 50.0	(50.0–100.0) / 17.5
SFO, %	10	72.5 / 75.0	(40.0–100.0) / 19.6
SCO, %	10	79.5 / 87.5	(20.0–100.0) / 24.3
SSO, %	10	7.0 / 0.0	(0.0–35.0) / 13.6
Wrist flexion°	16	53.4 / 60.0	(20.0–80.0) / 19.3
Wrist extension°	16	42.8 / 50.0	(0.0–70.0) / 23.7
Overall wrist movement°	16	96.3 / 112.5	(35.0–140.0) / 41.4
MCP 1 flexion°	15	54.3 / 60.0	(30.0–70.0) / 11.8
MCP 1 extension°	15	0.0 / 0.0	(− 30.0 to 20.0) 11.2
Overall MCP 1 movement°	15	55.0 / 60.0	(20.0–70.0) / 15.7
MCP 1 opposition (Karpandji 0–10)	15	7.4 / 9.0	(3.0–10.0) / 2.9
MCP 2 flexion°	13	70.4 / 90.0	(0.0–90.0) / 32.6
MCP 2 extension°	13	0.4 / 0.0	(− 15.0 to 10.0) / 5.9
Overall MCP 2 movement°	13	70.8 / 90.0	(0.0–100.0) / 34.3
MCP 3 flexion°	16	73.4 / 87.5	(0.0–90.0) / 29.0
MCP 3 extension°	16	2.5 / 0.0	(− 10.0 to 20.0) / 7.7
Overall MCP 3 movement°	16	75.9 / 90.0	(0.0–110.0) / 31.7
MCP 4 flexion°	15	72.3 / 90.0	(0.0–90.0) / 29.6
MCP 4 extension°	15	2.3 / 0.0	(− 5.0 to 20.0) / 7.3
Overall MCP 4 movement°	15	74.7 / 90.0	(0.0–110.0) / 31.6
MCP 5 flexion°	15	78.5 / 90.0	(0.0–95.0) / 25.5
MCP 5 extension°	15	3.8 / 0.0	(0.0–20.0) / 7.7
Overall MCP 5 movement°	15	82.3 / 90.0	(0.0–115.0) / 28.4
Grip strengths, Kg	11	25.0 / 24.0	(10.0–56.0) / 11.7
Grip strengths, %^**+**^	11	25.0 / 24.0	(33.3–96.4) / 19.0
Reported satisfaction, pts (1–4)*	11	1.4 / 1.0	(1.0–3.0) / 0.7

*Pts* points, *VAS* visual analog scale, *SFO* subjective functional outcome, *SCO* subjective cosmetic outcome, *SSO* subjective sensibility outcome, *MCP* metacarpophalangeal joint

^**+**^Compared to the healthy side. *Satisfaction rated (1) excellent (2) good (3) fair (4) unsatisfactory

## Discussion

The aim of the current study was to describe the mid-term results of free gracilis muscle flaps covered with split thickness skin grafts used in extensive soft tissue defects in the hand. Considerations have been made to range of motion of the wrist and finger joints, tendon gliding ability, as well as grip strength among patient satisfaction. Unlike in the fingers, where some conservative treatment strategies demonstrated promising clinical results [14], surgery is the treatment of choice in soft tissue defects of the hand. While dorsal defects of the hand are easier to cover and offer more surgical options, palmar defects are more difficult to treat surgically. Engelhardt et al. proposed to divide the palm of the hand even into three units each requiring a different soft tissue coverage [16]. Main aims of covering palmar defects are a sufficient supply of strength, high gliding capacity, high pliability with no impairment of range of motion, and low thickness of the flap [17, 18]. Nowadays, a large collection of microsurgical flaps is available. Sensate musculo- and fasciocutaneous flaps have the drawbacks of tissue mobility with shifting and bulkiness under stress, whereas fascial and thin muscle flaps often deal with adhesions [16, 19].

The gracilis muscle free flap is considered one of the standard flaps in reconstructive microsurgery owing to its low harvesting morbidity, ease of harvesting, consistent anatomy, and wide variety of potential applications. However, it has drawn criticism for its bulkiness and skin tethering [20].

In a survey comparing muscle and fasciocutaneous free flaps for lower limb reconstruction, Seyidova et al. discovered that patients who received a fasciocutaneous flap were more satisfied with the flap’s texture than those who received the muscle flap. Yet, regarding contour, resemblance to the contralateral side, flap bulkiness, color match, scarring, overall appearance, and donor-site outcomes, no significant changes between the two flaps were discovered [21].

The current study confirmed that the free gracilis flap does not always lead to an excessive bulkiness neither of the palm nor dorsal aspect of the hand. Even for palmarly, it represents a good solution because it does not lie flabby on the underlying tissue. Unlike fasciocutaneous flaps, it has the advantage of a shrinking but remaining still a pliable thin muscle flap. It is important to outline that the gracilis flap does not result in a shrinkage in length, but only in thickness, which is especially important when it comes to cover moving joints.

Furthermore, the current study evaluated reasons for complications after the use of gracilis muscle flaps. Although some complications occurred, all except one flap (94%) ultimately survived and healed in the investigated cohort. Furthermore, patients showed good to excellent clinical results. Thus, the gracilis muscle flap represents a satisfactory alternative to other flaps that are commonly used to cover soft tissue defects in the hand. In contrast to the gracilis muscle flap, the lateral arm flap is limited due to its short pedicle and its possible size [22]. In 29 patients, Schecker et al. documented the use of the ipsilateral lateral arm free flap with most defects being at the dorsum of the hand, resulting in a success rate of 96.5% [23]. The authors observed tenderness in the region of the lateral epicondyle if it is not covered with full thickness skin. Moreover, forearm numbness could arise from a loss of the lateral cutaneous nerve. Furthermore, 16% of patients in a study published by Ulusal et al. and 15% of the patients in a study published by Graham et al. needed debulking of the lateral arm flap in due course [23, 24], considering the radial and ulnar flap, the sacrifice of a main hand artery beside the unsatisfactory donor-site of the radial or ulnar flap [26]. The radial forearm flap’s biggest limitation is its donor-site morbidity, which is not only functional but also esthetic because the majority of donor sites necessitate skin grafting and there might be dysesthesia in the supply area of the superficial radial nerve [27]. Acceptable esthetic results are described using the posterior interosseus flap with reverse flow. However, besides sacrificing the posterior interosseous artery, its size is limited and low esthetic satisfaction is achieved in cases where skin grafting is necessary for the donor site [28]. Another free flap that offers good clinical results is the free serratus flap. Tee et al. described a modification of this flap calling it the serratus carpaccio flap. The authors included a thin layer of the serratus muscle leading to an easier harvest of the flap and compared outcome parameters with the serratus fascia flap. Patient outcome was equal in both groups in patients with soft tissue defects of the hand and foot [28].

The anterolateral thigh (ALT) flap offers a vascular pedicle extending up to 15 cm, which makes it suitable for performing arterial anastomosis beyond the injured area [29]. Despite its minimal donor-site morbidity, large available amount of skin, its versatility, and reliability, there is a major downside with the necessity of thinning the ALT flap resulting in potential flap necrosis [30, 31].

Over a three-year period, Hanasono et al. monitored anterolateral thigh flaps in 220 patients. They discovered that the lateral femoral cutaneous nerve distribution was numb in 84% of their patients [32]. This finding corresponds to the results of Fricke et al., who found the gracilis muscle flap to be superior to the ALT flap with regards to scar length and degree of numbness at the donor side [33]. In addition, the gracilis muscle flap is a denervated muscle flap, unlike the fasciocutaneous ALT flap, which will atrophy with time, potentially improving flap form and esthetics without the need for numerous secondary treatments [33].

Comparing the gracilis muscle flap to the thoracodorsal artery flap and the scapular–parascapular flap, no change of position during surgery is required, thus saving valuable operation time [34, 34].

Fasciocutaneous flaps are thin and pliable. However, when anastomosed to distal vessels, their transplant might result in substantial vascular mismatch and additionally necessitates the sacrifice of a peripheral artery.

Compared to the previously mentioned flaps, the gracilis muscle flap has numerous advantages. It is often being chosen due to its suitable size and low donor-site morbidity [7]. Moreover, it is easy to harvest, making it a popular flap even for non-experienced surgeons offering a broadly reconstructive applicability. Although the findings of the current study are promising, further studies are needed to confirm the results in larger patient collectives.

### Methodological considerations

Limitations of this study include the retrospective design with prospective follow-up of only 16 patients. The systematic collection of complete clinical data for all patients undergoing microvascular gracilis muscle flaps for covering defects in their hands at our institution is, however, a robust basis for the present study. Furthermore, all gracilis muscle flaps used in the hands were analyzed in the current study. No detailed subgroup analysis could be performed regarding the position of the flap in the hand (e.g., palmar and dorsal). However, with only 16 patients receiving a gracilis muscle flap at our institution, it is difficult to increase the power for meaningful subgroup analyses, although key findings are so obvious.

## Conclusions

Coverage of large defects on the hands with a gracilis muscle flap showed a survival rate of 94%. Patients showed good clinical outcomes with acceptable wrist movement and grip strength as well as high reported satisfaction rates. Compared to the use of fasciocutaneous free flaps, the pliability and the thinness especially on the palmar aspect of the hand are satisfactory. Hence, covering large defects of the hand with a gracilis muscle flap can be a successful procedure.

## Conflict of interest

None.

## Ethics approval

This study was approved by the local ethics committee (KEK Bern, Switzerland, BASEC-Nr. 2021–01346) and was carried out in accordance with the Declaration of Helsinki. All participants provided their written informed consent.

## Informed consent

All participants provided their written informed consent.

## Data Availability

All data is available on resonable request.
